# Rheumatoid arthritis associated with the occurrence, severity and extension of periodontitis: A case-control study

**DOI:** 10.4317/jced.57540

**Published:** 2021-04-01

**Authors:** Marcela-Faria Moura, Luís-Otávio-Miranda Cota, Adriana-Moreira Costa, Tarcília-Aparecida Silva, Fernando-Oliveira Costa

**Affiliations:** 1 Department of Dental Clinics, Oral Pathology, and Oral Surgery, School of Dentistry, Federal University of Minas Gerais, Belo Horizonte, Minas Gerais, Brazil; 2 Newton Paiva Institute, School of Dentistry, Belo Horizonte, Minas Gerais, Brazil

## Abstract

**Background:**

Emerging evidence pointed to a potential association between periodontitis (PE) and rheumatoid arthritis (RA), based on shared characteristics and similarities in risk factors, immunogenetics and pathways of tissue destruction. The aim of this study was to evaluate the potential association between RA and PE, as well as the influence of risk variables in this association.

**Material and Methods:**

The present case-control study comprised 471 individuals (157 cases with RA and 314 controls) that underwent a full-mouth periodontal examination. The association between risk variables and the occurrence of AR and PE were evaluated through univariate and multivariate logistic analysis.

**Results:**

Higher frequency (*p*<0.001), severity (*p*=0.006) and extension (*p*=0.018) of PE was observed among the cases when compared to controls. Variables retained in the final multivariate models for the occurrence of PE were: lower number of teeth, smoking, no use of dental floss, ≥4 daily toothbrushing and RA; for the occurrence of RA were: higher age, female gender, smoking, alcohol use and PE. It is important to stress that RA (OR=2.53; 95%CI 1.24–3.86; *p*<0.001) was retained in the model for PE, and PE (OR=3.12; 95%CI 1.47–4.26; *p*<0.001) was retained in the model for RA.

**Conclusions:**

The present study demonstrated a high frequency of PE among individuals with RA and an important association among the occurrence, severity and extension of PE and RA and smoking.

** Key words:**Case-control study, risk factors, periodontitis, rheumatoid arthritis.

## Introduction

Periodontitis (PE) is characterized by a microbiologically associated inflammation (1). Its pathogenesis is the outcome of complex interactions between periodontal pathogens and host immune response, having molecular pathways leading to the activation of host-derived proteinases and resulting in the migration of the junctional epithelium, subsequently destroying the periodontal attachment (2).

Several studies have demonstrated evidence of the association between PE and several systemic conditions, including diabetes, obesity, cardiovascular diseases, pregnancy disorders and rheumatoid arthritis (RA) (3,4). The underlying biological plausibility is based on the concept that PE inflammation and the periodontal microbiome contribute to the global burden of systemic inflammation at a level that affects the occurrence, severity and progression of other chronic inflammatory conditions (5).

RA is a chronic autoimmune disease that causes a breakdown of self-tolerance, chronic inflammation and debilitating joint destruction (6), compromising synovial fluids, joint cartilage and bone integrity (7). The etiology of RA remains uncertain, but the activity of periodontal pathogens has been linked to the production of RA auto-antibodies (8). RA is characterized by the presence of rheumatoid factor and anti-citrullinated protein / peptide antibodies (ACPAs) (9).

Some studies (10-17) pointed to a potential association between PE and RA, based on shared characteristics and similarities in risk factors, immunogenetics and pathways of tissue destruction (18,19). These diseases essentially differ, being RA an autoimmune disease while PE is an infectious disease (2).

A specific hypothesis suggests that certain oral bacteria can induce protein citrullination under the action of the enzyme peptidyl arginine deiminase, existing in both **Porphyromonas gingivalis** and inflammatory cells. Thus, **P. gingivalis** can play a crucial role in breaking immunological tolerance by inducing the citrullination of host proteins, converting them into autoantigens (19). These modified proteins, under a common immunogenic background, can be recognized by the immune system, triggering an inflammatory process that is associated with the clinical manifestations of both diseases. ACPAS are the most suitable biomarkers for tracking RA and are a common serological finding in RA and PE (19-21). ACPAs are associated with radiographically detectable damage and extra-articular manifestations, found years before the beginning of clinical RA (20).

A recent systematic review of observational studies revealed that, although most reported an association between RA and PE, others claim that this association may be related to biases in PE assessment and to the absence of treatment for confounding factors. In addition to conflicting data, the quantitative analysis showed a high heterogeneity among studies, leading to the need for further studies (9).

The present study focused on evaluating the periodontal condition and the clinical and epidemiological aspects of the association between PE and RA through a case-control study.

## Material and Methods

-Sampling strategy

The present case-control study comprised a convenience sample of 725 individuals, male and female, age ranging from 35 to 65 years old, with no antibiotic use for a 3-month period prior to the study entry and a minimum of 6 month since the last periodontal treatment. Individuals were selected from those who were under medical treatment at the Rheumatology Outpatient Clinic from the Hospital das Clínicas of the Federal University of Minas Gerais, Belo Horizonte – Brazil.

Sample size was determined through the Fleiss Method with continuity correction, considering a significance level of 0.05, 90% study power, and a 1:2 proportion between case and controls. Therefore, assuming an expected prevalence of PE of 40% among cases with AR and 25% among healthy controls, a number of 122 cases and 244 controls were determined to be necessary.

**Figure 1:**
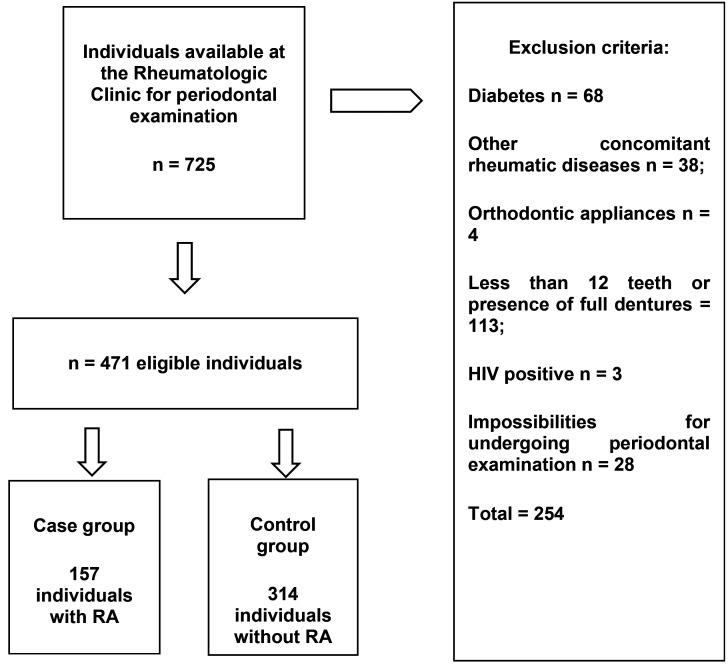
Sampling strategy and study sample.

During the period of data collection, from September 2018 to January 2020, 725 individuals were interviewed and available for periodontal examination. After applying the exclusion criteria, 254 individuals were excluded (diabetes n = 68; other concomitant rheumatic diseases n = 38; orthodontic appliances n = 4; less than 12 teeth or presence of full dentures = 113; HIV positive n = 3; impossibilities for undergoing periodontal examination n = 28), thus 471 individuals were examined and determined to be eligible for the study. Final sample comprised 157 individuals with RA (cases) and 314 without RA (controls). Control group was composed by appointment companions, relatives, and staff members from the study hospital unit, all of them without RA. Sampling strategy is shown in Figure 1.

The present study was approved by the Research Ethics Committee from the Federal University of Minas Gerais, Belo Horizonte – Brazil (CAAE#48355915300005149). All individuals provided written informed consent before enrolling in the study.

-Data collection

Interviews were performed through a structured questionnaire, recording the following data: gender, age, general health status, alcohol consumption (frequency / amount), smoking and oral hygiene habits. It was also collected from the medical records of all case individuals data on RA activity through the Disease Activity Score (DAS-28) (22). The most common medications used are methotrexate, vitamin D, calcium, folic acid, leflunomide e prednisone.

In relation to smoking, individuals were determined to be smokers if they had smoked ≥100 cigarettes throughout life and were still smoking during the study examination period; those who had smoked <100 cigarettes throughout life and were not smoking at the study examination period were determined to be non-smokers (23).

-Periodontal clinical examination

Periodontal clinical examination was performed with a manual periodontal probe. The following periodontal parameters were evaluated and recorded from all present teeth, except from third molars, at 4 sites (mesial, distal, buccal e lingual): (i) probing depth (PD); (ii) clinical attachment level (CAL); (iii) bleeding on probing (BOP) (iii).

-Intra and inter-examiner agreement

Periodontal examinations were performed in 12 individuals by 2 trained and expert periodontists (M.F.M and F.O.C). Intra and inner-examiner agreement for PD and CAL parameters revealed kappa values higher than 0.88 and intraclass correlation coefficient higher than 0.90.

-Periodontitis definition and staging

Individuals were classified according to periodontitis stages and defined as periodontitis cases from stage II: Stage II: ≥ 2 interproximal sites with CAL of 3 to 4 mm, PD ≤5 mm, horizontal bone loss up to the coronal third (15% to 33 %) and without tooth loss due to periodontitis. Total periodontitis were defined as the sum of stages II, III and IV, according to the criteria by Tonetti *et al.* (1).

-Rheumatoid arthritis diagnosis 

Diagnosis and severity of RA (early, moderate and severe stages) were determined by the rheumatologic medical team of the study hospital, according to the criteria stablished by the American College of Rheumatology (24). These include having at least 4 of the following characteristics for at least 6 weeks: morning joint stiffness lasting for at least 1 hour; arthritis in at least three joint areas; arthritis of hand joints and wrists, proximal interphalangeal joints (middle finger joint) and metacarpophalangeal joints (between fingers and hand); symmetrical arthritis (in the left and right wrist, for example); presence of rheumatoid nodules; presence of rheumatoid factor in the blood and radiographic changes: joint erosions or decalcifications located on hand and wrist radiographs. In addition, RA activity was assessed using the DAS-28 method (22) in stages of activity, i.e., remission, early, moderate and high activity.

-Statistical analysis

Groups were initially compared in relation to the following variables: gender, age, smoking (non-smokers / former-smokers and smokers), alcohol consumption, oral hygiene habits, DAS-28 scores and use of medications by the Chi-square and Mann-Whitney tests and Spearman correlation, when appropriate. Regarding the periodontal parameters (PD, CAL, BOP), the values per individual were obtained by the sum of the measures of all periodontal sites and expressed as means and / or percentages.

The distribution of independent variables by PE stages, odds ratio (OR) and their 95% confidence intervals (CI) were calculated. The effect of variables of interest on the occurrence of PE and RA was assessed using the multivariate logistic regression. The Generalized Estimation Equations method was used to obtain marginal logistic models that directly incorporate the correlation between the measures of the same sample unit. Variables with a *p*-value <0.25 in the bivariate analysis were then included in each multivariate model (non-cases (0) and cases (1)) and removed manually step by step until the likelihood ratio test indicated that no variable should be removed. All variables included in the final multivariate models were determined to be independent by assessing their collinearity. The quality of the models was determined by measures of sensitivity, specificity, area under the ROC (Receiver Operating Characteristic) curve, R² (Nagelkerke) and Hosmer-Lemeshow test. All analyzes were performed using statistical software R.

## Results

Sample comprised 471 individuals, being 145 male and 326 female, with mean age of 47.39±5.18 years old in the control group and of 54.1±11.45 years old in the case group. The frequency of alcohol use among controls and cases was 54.2% and 20.7%, respectively (*p*<0.001). Moreover, individuals with RA showed significantly higher mean age (*p*<0.001), smokers (*p*<0.001) and less use of dental flossing (<0.025) and less alcohol consumption (*p*<0.001). Brushing frequency did not show significant differences between the groups (Table 1).

**Table 1:**
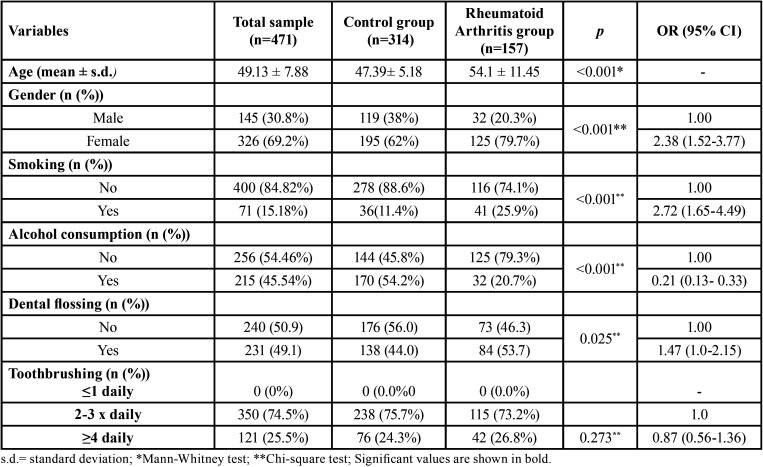
Characterization of the sample.

A high frequency of total PE (stages II + III + IV) was observed among cases (53.8%) when compared to controls (30.1%) (*p*<0.001). Individuals with RA presented a 2.64 higher chance of having PE (unadjusted OR = 2.64; 95%CI 1.78–3.94; *p*<0,001). In addition, the severity (*p* = 0.006) and extension (*p* = 0.018) of PE were higher among RA individuals (Table 2).

**Table 2:**
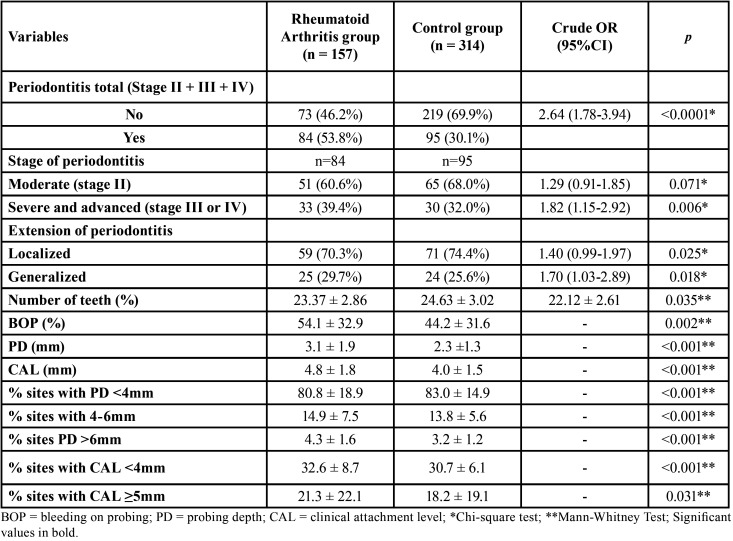
Periodontal clinical parameters of the sample.

Regarding periodontal clinical parameters, individuals with RA presented significantly worse parameters than individuals without RA, that is, lower number of teeth, higher average of sites with BOP, higher PD and CAL, higher percentage of sites with PD 4–6 mm and PD ≥6mm, higher percentage of sites with CAL ≥3mm and CAL ≥5mm (Table 2).

Table 3 shows the final logistic regression models and respective variables significantly associated with: (i) the occurrence of PE (model 1) – RA, lower number of teeth, smoking, no use of dental flossing and protective effect for ≥4 daily toothbrushing; (ii) the occurrence of RA (model 2) – PE, older age, female sex, smoking and a protective effect of alcohol use. It is noteworthy that PE (OR = 3.12; 95%CI 1.47–4.26; *p*<0.001) remained in the model for AR and AR (OR =2.53; 95%CI: 1.24- 3.86; *p*<0.001) remained in the model for PE.

**Table 3:**
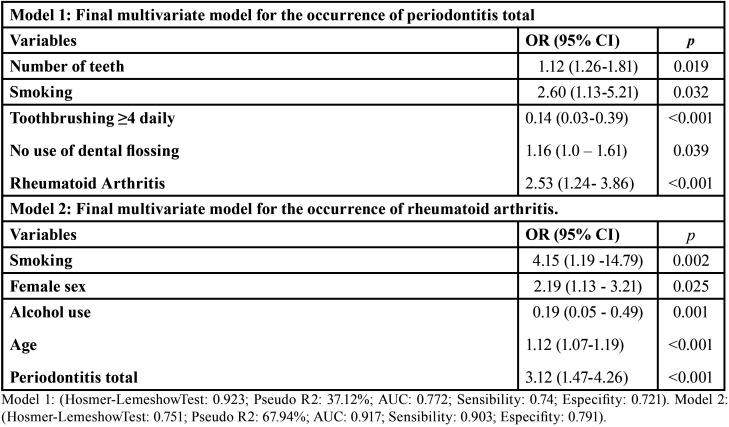
Final multivariate logistic models for the occurrence of periodontitis and rheumatoid arthritis.

RA activity by stages (remission, early, moderate and high activity) was assessed through the DAS-28 method and correlated with PE. However, there was no significant correlation between DAS-28 scores and periodontal clinical parameters of PD, CAL and BOP, occurrence of PE, as well as in relation to the severity of PE (*p*>0.05; data not shown).

The use of different medications such as methotrexate, vitamin D, calcium, folic acid, leflunomide and prednisone by individuals with RA did not shown significant differences in relation to the occurrence or the severity of PE (*p*>0.05; data not shown).

## Discussion

The present study showed a higher occurrence of PE in individuals with RA when compared to controls (OR=2.64; 95% CI: 1.78–3.94; *p*<0.001). In addition, greater severity and extension of PE were also observed. This association remained in the multivariate models for both PE and RA.

It is hypothesized that both conditions share common characteristics and pathogenic similarities in relation to immunogenetics and tissue destruction pathways (21). They are chronic inflammatory diseases that share mutual risk factors for susceptibility, such as HLA-DRBI alleles and smoking. In addition, the role of **P. gingivalis** and its enzyme PAD, that is capable of generating citrullinated epitopes that are recognized by ACPAs, has been proposed as a link between PE and RA. Recently, two isoforms of the peptidyl arginine deiminase family (PAD2 and PAD4) and citrullinated proteins have been identified in inflamed periodontal tissues, reinforcing the hypothesis that periodontal citrullination may play an important role in the etiopathogenesis of RA (19,21).

Observational studies have shown that patients with RA have an increased occurrence of PE compared to healthy individuals without RA (11,19,25-30). Additionally, the frequency of RA was higher in patients with PE compared to those without PE (14,15). However, findings from other population-based studies have failed to show any association (10,12,26,30) or showed a weak association (13) between these two conditions.

Potential frequent biases can be observed in several studies, such as the use of retrospective data obtained from medical records, small samples, indexes or radiographic exams to evaluate the occurrence of periodontitis, self-reported information about periodontal status and RA. Thus, additional studies that control these factors are necessary.

In the present study, individuals with RA had significantly worse periodontal status when compared to controls, presenting a higher average of sites with BOP, higher mean PD and CAL and lower number of teeth. In the study by Rosamma *et al.* (28) and Zhao *et al.* (29), worse clinical periodontal parameters were also observed among individuals with RA.

DAS-28 is the gold standard method to measure RA activity (22). In this present study, there was no significant correlation between DAS-28 scores and periodontal clinical parameters (PD, CAL and BOP) in relation to the occurrence and severity of PE. Different studies (28-30) have also found no association between rheumatoid disease activity and the occurrence and severity of PE. However, in the study by Choi *et al.* (31), BOP was correlated with DAS-28 scores, although no correlations for PD and CAL.

On the other hand, in the study De Smit *et al*. (27), individuals with RA and severe PE had higher DAS-28 scores when compared to individuals with RA and moderate PE or even without PE. The presence of PE was also associated with greater disease activity according to DAS-28 scores by Mikuls *et al*. (11) and Zhao *et al.* (29).

In a recent systematic review and meta-analysis, there was no substantial effect of RA on PD and CAL among individuals with PE when compared to controls. However, it was demonstrated that there is consistent evidence suggesting that PE is associated with worse clinical activity of RA, as assessed by DAS-28 scores, whereas individuals with RA do not worsen clinical parameters of PE. It is important to note that a high heterogeneity was observed among the 6 meta-analyzed studies (26).

Depending on the activity of RA and on the individual response, the use of several medications is necessary to minimize tissue damage and control the disease. In past decades, it has been suggested that the use of anti-inflammatory drugs and corticoids could decrease the severity and occurrence of PE (30).

However, this issue was not sustained over the years. As few studies have approached this topic, the present study investigated the most used medications among individuals with RA such as methotrexate, vitamin D, calcium, folic acid and prednisone. Findings showed that the use of any of the mentioned drugs did not infer significant differences in relation to the occurrence and severity of PE. Ziebolz *et al.* (30) reported that drugs used to treat RA may be associated with periodontal inflammation, but without differences in the severity of PE.

The multivariate analysis for the occurrence of PE retained the following variables in the final logistic model: RA, lower number of teeth, smoking, no dental flossing and a protective effect for ≥4 daily toothbrushing. Corroborating previous findings, variables such as smoking and worse oral hygiene habits can contribute to the activation of systemic triggers for a prolonged period, leading to immunological changes (exacerbation in the expression of cytokines), as well as endocrine and behavioral disorders, that can predispose to greater susceptibility to both diseases (3,19).

Tooth loss is considered the final outcome of oral diseases that affect periodontal and dental tissues, affecting the oral health-related quality of life. In this present study, individuals with RA presented significantly lower number of teeth than controls. Similar results have been previously reported (19,28,29).

The multivariate analysis for the occurrence of RA retained the following variables in the final logistic model: PE, smoking, female gender, older age, and a protective effect of lower alcohol use. Smoking, female sex and age are known to be strong risk factors for the occurrence of RA. According to Hussain *et al.* (26), the reduction in the frequency of alcohol use revealed significant improvements in pain and quality of life in individuals with RA.

It is important to highlight that in both multivariate models, PE (OR = 3.12; 95% CI: 1.47–4.26; *p*<0.001) remained in the model for AR and AR (OR = 2.53; 95% CI: 1.24–3.86; *p*<0.001) remained in the model for PE. This reinforces the association between these 2 conditions. In addition, the presence of smoking in both models also reinforces the hypothesis that common risk and susceptibility factors are shared between them.

Some limitations must be attributed to the present study. The convenience sample may have some impact on the external validity of the results. Due to temporality, the case-control study does not detect any temporal influence between RA and PE, as well as direction of the association cannot be clearly established. Another issue is that this study design is susceptible to selection bias, since exposure, disease occurrence and its outcomes is recorded at the moment the patient is recruited for the study. However, in order to minimize these factors, all individuals in sample of the present study were recruited from a single center with homogeneous characteristics.

On the other hand, advantages can also be cited for the present study as: (i) the high number of individuals in the sample, increasing the statistical power of the study; (ii) the diagnosis of RA by specialized doctors; (iii) the full-mouth periodontal examination with a robust periodontitis definition, since it is recognized that the quality of the periodontal data and the criteria for PE definition can strongly impact the results of the association studies (3).

Future studies with different samples and prospective designs, including microbiological and immunological analyzes, would be of paramount importance to provide more accurate conclusions about the periodontal status of individuals with RA and the association between PE and RA.

The present study reported a higher occurrence, severity and extension of PE in individuals with RA. A strong association between RA and PE was observed after adjusting for important confounding variables in multivariate models, including smoking as a common risk factor shared between both conditions.
